# Glycemic Control Status and Long-Term Clinical Outcomes in Diabetic Chronic Total Occlusion Patients: An Observational Study

**DOI:** 10.1155/2021/5565987

**Published:** 2021-04-21

**Authors:** Xuehui Zhang, Maoxiao Nie, Xue Chen, Zhe Liang, Quanming Zhao

**Affiliations:** ^1^Beijing Anzhen Hospital, Capital Medical University, Beijing Institute of Heart, Lung and Blood Vessel Diseases, The Key Laboratory of Remodelling-Related Cardiovascular Diseases, Department of Cardiology, 2 Anzhen Road, Chaoyang District, Beijing 100029, China; ^2^Beijing Friendship Hospital, Capital Medical University, Beijing 100029, China

## Abstract

**Background:**

Whether good glycemic control can result in clinical benefits for diabetic chronic total occlusion (CTO) patients is still a matter of debate.

**Methods:**

We studied 1029 diabetic CTO patients. Based on one-year glycosylated hemoglobin A (HbA1c) levels, we assigned the patients into 2 groups: HbA1c<7% group (*n* = 448) and HbA1c ≥ 7% group (*n* = 581). We further subdivided the patients into the successful CTO revascularization (CTO-SR) and nonsuccessful CTO revascularization (CTO-NSR) groups. Kaplan–Meier analysis and Cox regression before and after propensity score matching were used to compare major adverse cardiovascular events (MACE) and other endpoints.

**Results:**

There were no significant differences between the groups in terms of most endpoints in the overall patients. After propensity score-matched analysis, patients with HbA1c < 7.0 tended to be superior in terms of MACE, which was mainly attributed to repeat revascularization but the other endpoints. Furthermore, the benefit of the HbA1c < 7 group was more prominent among patients with CTO-NSR in terms of MACE, repeat revascularization, and target vessel revascularization (TVR); and the improvement of the HbAc1 < 7 group was more prominent among patients without chronic heart failure (CHF) (*P*=0.027).

**Conclusions:**

HbA1c < 7.0 was associated with a reduced incidence of MACE, which was mainly attributed to a reduction in repeat revascularization. Good glycemic control can improve diabetic CTO patients' clinical prognosis, especially in CTO-NSR patients.

## 1. Background

Diabetes is common in patients with chronic total occlusion (CTO) [1]. Moreover, patients with diabetes suffer more adverse cardiovascular outcomes than patients without diabetes [2, 3]. We sought to assess if a well-controlled glycemic level had a beneficial effect on improving clinical prognosis. Previous studies consistently reported that intensive glycemic control could reduce microvascular complications [4]. However, when considering macrovascular complications, the benefits of good glycemic control are still under debate [5, 6].

CTO is a type of macrovascular diseases that is characterized by severe coronary artery disease and late-stage atherosclerosis [7]. However, microcirculation is also involved in the progression of CTO. Collateral circulation, a form of microcirculation [8], also plays an important role in irrigating viable myocardium of the CTO territory and indeed influences clinical prognosis [7, 9, 10]. Unfortunately, collateral circulation is impaired in diabetic patients [11, 12]. We wondered if the beneficial effects of good glycemic control on microvascular vessels were also observed in collateral circulation and, hence, subsequently benefited CTO patients. To date, no study has focused on this issue.

Therefore, we conducted this retrospective cohort study that enrolled diabetic patients with stable CTO. We wanted to explore whether glycemic control results in clinical benefits for diabetic CTO patients.

## 2. Materials and Methods

### 2.1. Study Population

Between January 2007 and December 2017, a total of 2502 diabetic CTO patients were consecutively enrolled in a retrospective cohort study. The present study further extracted patients with glycosylated hemoglobin A (HbA1c) data at the one-year follow-up. The Clinical Research Ethics Committee of the Beijing Anzhen Hospital approved the protocol (No.: 2018008X). The inclusion criteria were as follows: (1) diabetic patients with one main stem CTO (American Heart Association segment maps 1, 2, 3, 6, 7, 8, 11, and 13) that was diagnosed by coronary angiography; (2) patients with manifestations of symptomatic stable angina or silent ischemia; and (3) patients with HbA1c data at the one-year (9 months to 15 months) follow-up. The exclusion criteria were as follows: (1) coronary artery bypass grafting (CABG) history; (2) left main coronary artery stenoses ≥50%; (3) a history of acute myocardial infarction (MI) due to a non-CTO vessel within one month; and (4) tumor or other diseases that might confound interesting endpoints. Finally, a total of 1029 patients were included.

**Figure 1:**
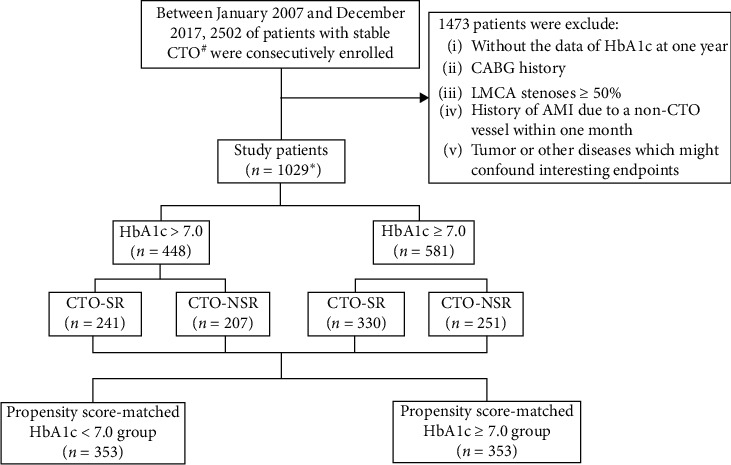
Study scheme. AMI: acute myocardial infarction; CABG: coronary artery bypass grafting; CTO: chronic total occlusion; LMCA: left main coronary artery; CTO-SR: successful CTO revascularization, a successful revascularization for the CTO vessel by percutaneous coronary intervention (PCI) or coronary artery bypass grafting (CABG); CTO-NSR: nonsuccessful CTO revascularization, patients failed CTO revascularization procedures or failed to try CTO revascularization (only took medicine). #Patients with manifestations of symptomatic stable angina or silent ischemia. ^*∗*^A total of 989 (96.11%) patients completed the follow-up process.

The enrolled patients were assigned to different groups according to HbA1c levels at the one-year follow-up: HbA1c < 7% group and HbA1c ≥ 7% group. Furthermore, considering that the occlusion status of the CTO vessel may influence the outcomes, we subdivided patients into two subgroups: patients with successful CTO revascularization (CTO-SR) and patients with nonsuccessful CTO revascularization (CTO-NSR). CTO-SR was defined as successful revascularization of the CTO vessel by percutaneous coronary intervention (PCI) or CABG. Patients who underwent failed CTO revascularization procedures or failed to try CTO revascularization (only taking medicine) were considered CTO-NSR (Figure 1).

### 2.2. Procedures

The baseline variables (age, sex, prior clinical history, inspection, and laboratory information among other factors) and endpoints of interest were extracted from the hospital information system (HIS) by researchers who were previously trained to ensure consistency.

A minimum of 12 months of follow-up was predefined. Phone call was the preferred method of follow-up. For patients who had records of rehospitalization at Beijing Anzhen Hospital, the necessary data were also collected from the HIS. All the endpoints and relevant variables were evaluated by an independent adjudication board blinded to the patient groups.

### 2.3. Outcomes and Other Variable Definitions

Coronary CTO was defined as total occlusion of the coronary artery (thrombolysis in myocardial infarction (TIMI) grade 0 flow) with a duration ≥ 3 months [13, 14]. The occlusion duration was calculated based on previous angiography, the occurrence of myocardial infarction, or the first episode of angina. Stenosis > 50% detected by coronary angiogram was considered diseased. Patients who failed to exhibit any clinical manifestations were artificially considered to meet our criteria. Diabetes was defined based on (1) a prior diagnosis of diabetes or use of glucose-lowering medicine before hospitalization and (2) a new diagnosis of diabetes (fasting blood glucose level ≥ 7.0 mmol/L or glucose level after a meal (two hours) ≥ 11.1 mmol/L, which was detected on at least 2 occasions) [3].

The primary endpoint was major adverse cardiovascular events (MACE), which was a composite of cardiac death, repeat revascularization, and repeat nonfatal myocardial infarction (MI). The definition of cardiac death followed the Academic Research Consortium (ARC) [15]: a death of cardiac, unknown or unwitnessed cause. The definition of repeat MI was based on the third universal definition of MI [16]: a composite of persistent ischemic angina symptoms, electrocardiogram, and elevations in myocardial injury biomarkers. The repeat nonfatal MI was used as our endpoint. Repeat revascularization was predefined as unplanned revascularization (by PCI or CABG) to the target vessel (CTO vessel) or other nontarget vessels. Other endpoints included target vessel revascularization (TVR) and all-cause death. TVR was predefined as an unscheduled revascularization (PCI or CABG) of the CTO vessel. All-cause death was predefined as a death due to any cause.

### 2.4. Statistical Analysis

Continuous variables with normal distributions are presented as the means ± SDs and were assessed by Student's *t*-test. Variables without normal distributions are shown as medians with interquartile ranges, and differences between the groups were compared using the Mann–Whitney *U* test. Categorical data are presented as numbers and percentages and were analyzed by the chi-square test or Fisher's exact test, where applicable.

The Kaplan–Meier method was used to construct survival curves of all the clinical outcomes. Comparisons were performed using log-rank tests. Unadjusted hazard ratios (HRs) were generated using the univariate Cox regression model. Covariates that were either clinically relevant or statistically significant (*P* < 0.2) were included in the multivariate Cox regression model. In summary, adjusted HRs were based on sex, age, chronic kidney disease (CKD), peripheral vascular disease (PVD), systolic heart failure, left ventricular ejection fraction (LVEF), regional wall motion abnormalities (RWMA), single-vessel disease, triple-vessel disease, left anterior descending artery-chronic total occlusion (LAD-CTO), left circumflex chronic total occlusion (LCX-CTO), Rentrop grade ≥ 2, and percutaneous coronary intervention with taxus and cardiac surgery (SYNTAX) score.

Propensity score-matched analysis was performed to further balance potential bias. All the baseline variables listed in Table 1 (except for retrograde approach and death during hospitalization) were included in the nonparsimonious model. A 1 : 1 ratio using the nearest-neighbor algorithm (caliper value = 0.02) was applied. Absolute standardized differences (ASDs) were applied to assess the imbalance of all the variables. A relatively good match was defined as ASDs less than 10.0%. After propensity matched analysis, the baseline characteristics listed in Table 2 were analyzed by using Student's *t*-test, Mann–Whitney *U* test, Fisher's exact test, or chi-square test, where applicable. Clinical outcomes were also reanalyzed by using the Kaplan–Meier method. The univariate Cox proportional hazard regression model was applied to calculate the HRs.

Furthermore, considering that the occlusion status of the CTO vessel may influence the outcomes, we performed a subgroup analysis based on the CTO occlusion status: CTO-SR and CTO-NSR.

Other post hoc subgroup analyses were performed according to age (<60 years old/≥60 years old), sex (male/female), prior MI (yes/no), chronic heart failure (yes/no), triple-vessel disease (yes/no), Rentrop grade ≥ 2 (yes/no), and SYNTAX score (<22/≥22), which were performed using a Cox regression model. The covariates included in the model were HbA1c, CTO-SR, age, PVD, history of prior myocardial infarction, heart failure (HF), LAD disease, sex, Rentrop grade, prior PCI, low-density lipoprotein (LDL), single-vessel disease, multivessel disease, and SYNTAX score. Moreover, post hoc subgroup analysis was only conducted on the primary endpoint, namely MACE.

All the analyses were conducted using SPSS 24.0 (SPSS Inc., Chicago, Illinois, USA) and Stata 14.0 (Stata, College Station, TX, USA). A two-tailed *P* value ≤ 0.05 was considered statistically significant.

## 3. Results

### 3.1. Baseline and Angiographic Characteristics (Total Population)

One year (9 months to 15 months) after enrollment, HbA1c information was successfully obtained for a total of 1029 patients: HbA1 < 7.0 (*n* = 448) versus HbA1c ≥ 7.0 (*n* = 581). The baseline characteristics are listed in Table 1. In summary, the patients with HbA1c ≥ 7.0 had higher baseline fasting blood glucose levels; higher SYNTAX scores; and higher prevalence of PVD, baseline HbA1c ≥ 7.0, and insulin uptake; however, these patients had lower prevalence of hypertension, dyslipidemia, prior stroke, hyperuricemia, single-vessel disease, sulfonylurea uptake, and thiazolidinedione uptake.

### 3.2. Clinical Endpoints in the Overall Population

**Figure 2:**
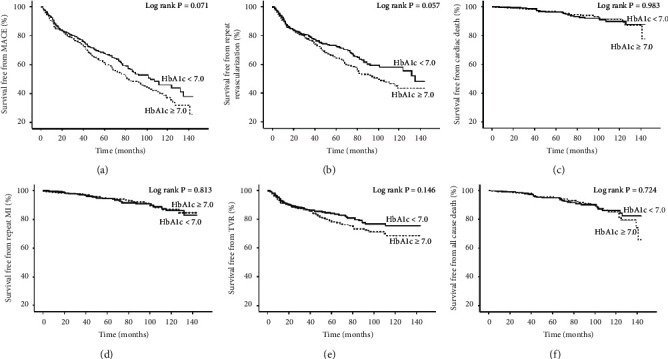
Kaplan–Meier cumulative event curves of MACE and secondary endpoints in the overall population. HbA1c: glycosylated hemoglobin A; MACE: major adverse cardiovascular events, which was a composite of cardiac death, repeat revascularization, and repeat nonfatal MI; TVR: target vessel revascularization; MI: myocardial infarction.

A total of 989 (96.11%) patients completed the follow-up process. After a median period of 44.00 (interquartile range (IQR): 20.00–67.00) months, MACE was observed in 157 (35.0%) patients in the HbA1c < 7.0 group and 223 (38.4%) patients in the HbA1c ≥ 7.0 group (unadjusted HR: 1.206, 95% CI: 0.983–1.479; adjusted HR: 1.194, 95% CI: 0.968–1.471). Cox regression analysis demonstrated no significant difference between the groups in terms of cardiac death, repeat nonfatal MI, all-cause death, and TVR. When considering repeat revascularization, after multivariate Cox regression analysis, the patients with HbA1c ≥ 7.0 suffered a higher risk (unadjusted HR: 1.238, 95% CI: 0.993–1.544; adjusted HR: 1.257, 95% CI: 1.003–1.576) than the patients with HbA1c < 7.0 (Figure 2).

### 3.3. Propensity-Matched Population

**Figure 3:**
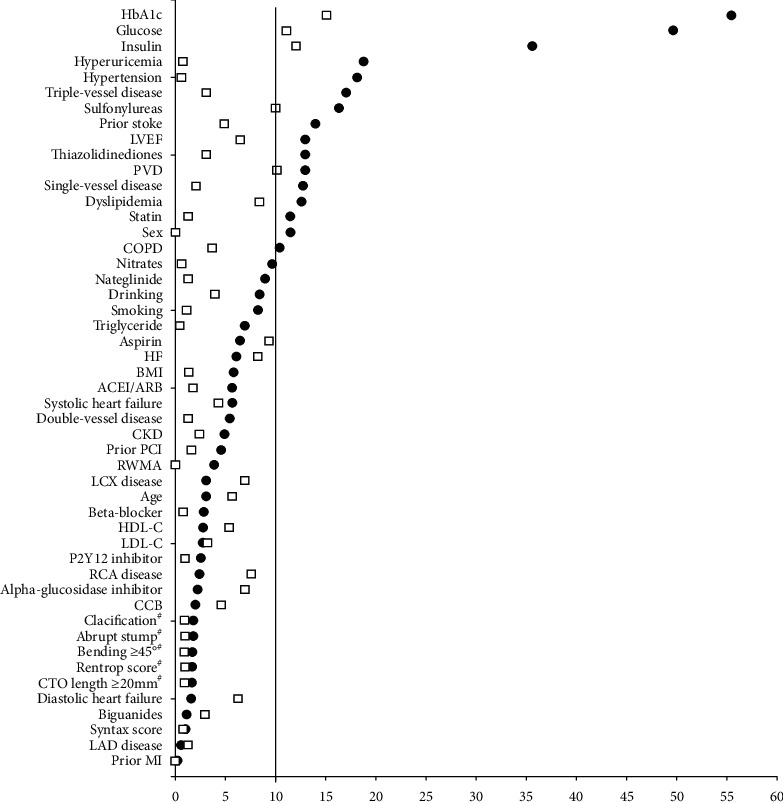
Standardized differences before and after propensity score match. Absolute standardized difference less than 10% indicates match well. CKD: chronic kidney disease; COPD: chronic obstructive pulmonary disease; PVD: peripheral vascular disease; MI: myocardial infarction; PCI: percutaneous transluminal coronary intervention; HF: heart failure; BMI: body mass index; ACEI/ARB: angiotensin-converting enzyme inhibitor/angiotensin-receptor blocker; CCB: calcium-channel blocker; HDL-C: high-density lipoprotein cholesterol; LDL-C: low-density lipoprotein cholesterol; LAD: left anterior descending; LCX: left circumflex; RCA: right coronary artery; LVEF: left ventricular ejection fraction; RWMA: regional wall motion abnormality. #Cine angiogram records got from 906 (88.05%) individuals.

**Figure 4:**
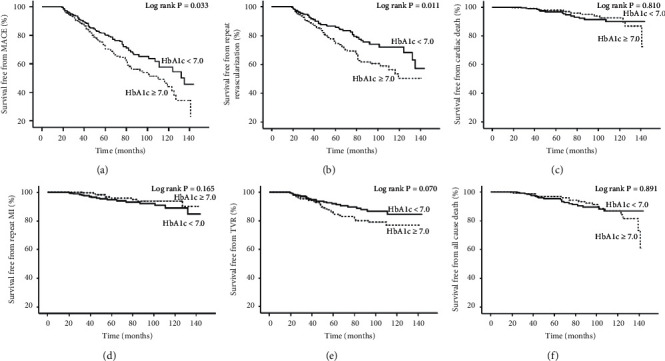
Kaplan–Meier cumulative event curves of MACE and secondary endpoints in the propensity score-matched population. HbA1c: glycosylated hemoglobin A; MACE: major adverse cardiovascular events, which was a composite of cardiac death, repeat revascularization, and repeat nonfatal MI; TVR: target vessel revascularization; MI: myocardial infarction.

After propensity matched analysis, 353 patients with HbA1c < 7.0 were matched with 353 patients with HbA1c ≥ 7.0, and the ASDs were all less than 10.0%, indicating that the patients with HbA1c < 7.0 or HbA1c ≥ 7.0 were well matched. Additionally, after reanalyzing the baseline variables, we found that they were all comparable except for baseline HbA1c (*P*=0.002) (Table 2 and Figure 3). Regarding clinical endpoints, Cox regression analysis demonstrated that the patients with HbA1c < 7.0 tended to be superior to those with HbA1c ≥ 7.0 in terms of MACE (unadjusted HR: 1.422, 95% CI: 1.027–1.970; adjusted HR: 1.531, 95% CI: 1.009–2.149), which was mainly attributed to repeat revascularization (unadjusted HR: 1.618, 95% CI: 1.111–2.356; adjusted HR: 1.828, 95% CI: 1.238–2.698). However, there were no significant differences between the 2 groups in cardiac death (unadjusted HR: 0.913, 95% CI: 0.434–1.921; adjusted HR: 0.717, 95% CI: 0.324–1.584), repeat nonfatal MI (unadjusted HR: 0.582, 95% CI: 0.268–1.261; adjusted HR: 0.513, 95% CI: 0.235–1.119), all-cause death (unadjusted HR: 1.044, 95% CI: 0.561–1.943; adjusted HR: 0.878, 95% CI: 0.457–1.687), and TVR (unadjusted HR: 1.595, 95% CI: 0.957–2.657; adjusted HR: 1.668, 95% CI: 0.994–2.796) (Table 3 and Figure 4).

### 3.4. Subgroup Analysis

After propensity matched analysis, we conducted various subgroup analyses to evaluate the association between HbA1c levels and the primary endpoint. The effect of blood glucose control was same among subgroups, regardless of baseline HbA1c. We found that the benefit of the HbA1c < 7 group was more prominent among patients with CTO-NSR than among patients with CTO-SR. HbA1c < 7 was significantly associated with improvement in terms of MACE (unadjusted HR: 1.566, 95% CI: 0.996–2.462; adjusted HR: 1.826, 95% CI: 1.112–2.999), repeat revascularization (unadjusted HR: 1.627, 95% CI: 0.969–2.733; adjusted HR: 1.906, 95% CI: 1.091–3.330), and TVR (unadjusted HR: 1.995, 95% CI: 0.987–4.034; adjusted HR: 2.194, 95% CI: 1.059–4.548). We also found that HbA1c < 7 correlated with increased risk for repeat nonfatal MI (unadjusted HR: 0.351, 95% CI: 0.112–1.103; adjusted HR: 0.242, 95% CI: 0.072–0.817) in the CTO-NSR subgroup (Table 4). The successful CTO revascularization was composed of the PCI subgroup (*n* = 269, 70% of the CTO-SR group) and the CABG subgroup (*n* = 117, 30%). Therefore, subgroup analyses were further performed in the PCI group and CABG group. We found that the benefit of the HbA1c < 7 group was more prominent among patients with PCI than among patients with CABG; and HbA1c < 7 was associated with improvement of MACE (*n* = 42, 30% vs. n = 57, 44%, *P*=0.019) in the PCI group. However, no significant difference was observed in terms of MACE among patients with GABG (*P*=0.406).

**Figure 5:**
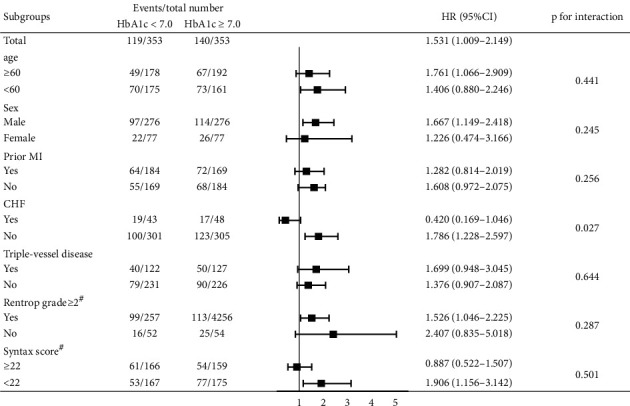
Comparative adjusted hazard ratios of the primary outs between the HbA1c < 7.0 group and the HbA1c ≥ 7.0 group for each subgroup in the propensity score-matched population. HR: hazard ratio; CI: conference interval; CHF: chronic heart failure; MI: myocardial infarction. #Cine angiogram records got from 906 (88.05%) individuals.

Other post hoc subgroup analyses were performed based on age (<60 years old/≥60 years old), sex (male/female), prior MI (yes/no), chronic heart failure (yes/no), triple-vessel disease (yes/no), Rentrop grade ≥ 2 (yes/no), and SYNTAX score (<22/≥22) in propensity score-matched population. The improvement of the HbAc1<7 group was more prominent among patients without chronic heart failure than among patients with chronic heart failure (Figure 5). Various subgroups, except for that with chronic heart failure, exhibited similar effects.

## 4. Discussion

### 4.1. Main Findings

In this retrospective cohort study, we enrolled 1029 diabetic patients with stable CTO. Glycemic control was reflected by the HbA1c level detected one year after enrollment. After a long-term follow-up, we observed that [1] in the overall population, there were no significant difference in the rate of primary endpoint except repeat revascularization. [2] After propensity matched analysis, patients with HbA1c ≥ 7.0 tended to suffer a higher risk of MACE than those with HbA1c < 7.0, which was mainly attributed to repeat revascularization; and a well-controlled glucose (HbA1c < 7.0) resulted in more substantial benefits for CTO-NSR patients in terms of MACE, repeat revascularization, and TVR. The results were different for CTO-SR patients. We found that the benefit of the HbA1c < 7 group was more prominent among patients with PCI than among patients with CABG. We think that the differences of the results before and after propensity score matching were the result of strong selection bias, as patients with poorly controlled HbA1c likely had many differences than those with controlled HbA1c as shown in Table 1. This article will focus on the results after propensity score matching and adjustment because propensity score match (353 pairs) corrected for differences in baseline differences.

Diabetes is considered equivalent to coronary artery disease due to its poor clinical outcomes [17]. In the CTO population, the prevalence of diabetes was as high as 34%–40% [18]. CTO patients with diabetes suffered poorer clinical outcomes than CTO patients without diabetes [19]. A few studies have demonstrated that hyperglycemia can result in an abnormal immune response, vascular inflammation, endothelial dysfunction, thrombosis, myocardial microangiopathy, and collateral circulation decreases, and excessive protein glycation end product formation and oxidative stress activation may be two primary mechanisms [6, 11].

However, whether glycemic control benefits diabetic CTO patients is unclear. Indirect evidence could be obtained from previous studies that focused on glycemic control and cardiovascular complications. The VADT (Veterans Affairs Diabetes Trial) [20, 21] enrolled 1791 military veterans. After a follow-up of 5.6 years, the study found that intensive glucose control (HbA1c approximately 7.0%) failed to affect the incidence of cardiovascular events, microvascular complications, and death. Similar results were also reported by the ACCORD trial and the ADVANCE trial [20, 22, 23]. However, the majority of patients enrolled in these three studies were patients without prior cardiovascular events. In the present study, we enrolled only CTO patients, who present with severe atherosclerosis. We demonstrated that patients with HbA1c < 7.0 were superior to patients with HbA1c ≥ 7.0 in terms of MACE, especially in the CTO-NSR subgroup. Our results were consistent with professor Hwang and colleagues [20], who studied 980 diabetic patients undergoing percutaneous coronary intervention and demonstrated that HbA1c < 7.0 (measured two years after PCI) was associated with a lower incidence of major adverse cardiac and cerebrovascular events (MACCE). However, the EXAMINE (Examination of Cardiovascular Outcomes: Alogliptin vs. Standard Care in Patients with Type 2 Diabetes Mellitus and Acute Coronary Syndrome) trial [9] reported an opposite outcome. A possible explanation for the different results is the different definitions of the standard of antidiabetic treatment and the different enrollment criteria or baseline characteristics of the subjects.

Importantly another point that should be emphasized is that vascular complications are not caused by hyperglycemia alone, but hypoglycemia is associated with an increased incidence of cardiovascular events [6, 24]. Three studies, including ADVANCE, ACCORD, and VADT, showed that hypoglycemia was associated with higher mortality rates than standard glycemic levels [25]. Currie et al. reported that type 2 diabetes mellitus (DM) patients with hypoglycemia had increased all-cause deaths and cardiac events compared with DM patients with standard glycemic levels [26]. These results were the same as those in the study by E. Marchionni, which showed that inappropriate hypoglycemia significantly increased the incidence of cardiovascular death in the intensive treatment group [27, 28]. Therefore, determining the optimal strategy for glycemic control in diabetic CTO patients has important clinical implications.

In our retrospective cohort study, to further examine the relationship between glycemic control and clinical outcomes, we selected HbA1c levels measured 1 year after enrollment, based on which the study population was divided into 2 groups: HbA1c < 7 and HbA1c ≥ 7 groups. Favorable effects were observed in patients with HbA1c < 7, and the incidence of MACE was lower in these patients than in patients with HbA1c ≥ 7; these results were mainly attributed to the decrease in repeat revascularization. In the subgroup analysis, strong benefits were observed in CTO-NSR patients in terms of MACE, repeat revascularization, and TVR. Taken together, our results suggest that good glycemic control may improve clinical outcomes in CTO patients with DM, especially CTO-NSR patients. We think that our study provides crucial new information about the target range for glycemic control in diabetic CTO patients.

To date, there have been few studies on the association between CTO in diabetes patients and adverse clinical outcomes. Abdulla et al. reported that, for diabetes patients with coronary heart disease, the presence of CTO of coronary arteries increases the risk of death in patients receiving medical therapy alone but may not increase the risk of death in patients treated with revascularization [18]. A previous study of CTO PCI in diabetes patients was performed by Bimmer, who reported reduced mortality of diabetes patients after successful CTO PCI [19]. However, in the present study, CTO-NSR patients benefited the most from well-controlled glucose (HbA1c < 7.0) in terms of MACE, repeat revascularization, and TVR. These benefits were not observed in CTO-SR patients. We think that these results may be explained by well-developed collaterals.

In CTO lesions, the normal coronary blood flow is completely occluded, and the majority of patients develop compensating vascular collateralization to supply ischemic distal tissue [7, 10]. Vascular collateralization is a response to slow progressive stenosis; given the prolonged duration of stenosis formation, blood flow is redirected into pre-existing collateral arteries bypassing the occluded artery [9]. For CTO-NSR patients, the downstream, postobstruction coronary artery segments depend entirely on collateral blood flow [10]. A previous study found that patients with well-developed collaterals have higher rates of survival and lower risk of cardiac death at 5 years than patients with poorly developed collaterals [10]. Similarly, some clinical data suggest that collateral blood flow can protect the myocardium of patients with CTO, for example, by reducing transmural myocardial ischemia [29, 30]. These results suggested that the degree of vascular collateralization may be significantly related to CTO patient outcomes. However, our study found that CTO-NSR patients benefited the most from well-controlled glucose (HbA1c < 7.0) in terms of MACE, but these benefits were not observed in CTO-SR patients. A possible explanation is that collaterals regress to a greater extent post-SR in CTO-SR patients.

The present study demonstrated a significant reduction in MACE in patients with HbA1c < 7.0, which was mainly attributed to a decrease in repeat revascularization. Although the use of second-generation everolimus-eluting stents (EES) improves treatment efforts of CAD after PCI, patients with diabetes mellitus (DM) have a 2–4 times higher risk compared with patients without DM in terms of rate of in-stent restenosis [2, 3]. The available evidence shows that chronic hyperglycemia can lead to vascular endothelial cell damage, with resultant abnormal vasodilation and vasoconstriction functions, excessive extracellular matrix formation, and promoted cellular proliferation, which in turn may lead to restenosis and TVR after PCI [31, 32]. DM itself can cause excessive thickening of the vascular intima and is the primary risk factor for higher stent restenosis event rates [33]. These findings are supported by some studies by Moussa et al., Jiménez-Quevedo et al., and Nobuyoshi Tanaka et al. These studies show that patients with DM have more frequent stent strut coverage, thicker neointima, and higher neointimal hyperplasia compared with patients without DM after drug-eluting stent implantation [33–35]. Therefore, a reduction in repeat revascularization is significantly associated with an improved rate of cardiovascular accidents in diabetic patients, and glucose control may be an important factor in determining the appropriate treatment strategy.

Revascularization of CTOs was accomplished by CABG or PCI with drug-eluting stent, and each revascularization strategy was selected as a treatment option by the patient and the attending physician based on the pathological characteristics and basic state of the patient. We therefore believe that PCI and CABG should be considered one unit, which is more consistent with the characteristics of real-world research. However, the benefit of well-controlled glucose (HbA1c < 7.0) was more prominent among patients with PCI than among patients with CABG, which should also be considered. Therefore, there is a need to pay more attention to blood glucose control in patients with PCI than in patients with CABG.

In the present study, the benefit of well-controlled glucose (HbA1c < 7.0) was more prominent among patients without chronic heart failure than among patients with chronic heart failure. Therefore, reducing the incidence rates of heart failure or improving cardiac function may play an important role in improving the rate of primary endpoint event in diabetic CTO patients, and reasonable glycemic control may be the main treatment method in this respect.

### 4.2. Limitations

There are several limitations in this study. First, the study was a nonrandomized, retrospective, and observational, which may weaken the statistical power of the conclusions due to confounding factors. Second, propensity score-matched analysis cannot correct for all the possible and unmeasured variables, which provide weaker evidence compared with randomized controlled trials. Third, study population are overall small numbers and may limit the influence of this work.

## 5. Conclusions

Patients with HbA1c < 7.0 tended to suffer a lower risk of MACE than those with HbA1c ≥ 7.0, which was mainly attributed to a reduction in repeat revascularization. Our data suggest that good glycemic control (HbA1c < 7.0) can result in clinical benefits for diabetic CTO patients, especially for CTO-NSR patients.

## Figures and Tables

**Table 1:** Baseline characteristics in the overall population.

Clinical characteristics	HbA1c < 7.0 (*n* = 448)	HbA1c ≥ 7.0 (*n* = 581)	*P* value
Age (year)	59.39 ± 9.66	59.68 ± 9.57	0.636
Male	354 (79.0)	431 (74.2)	0.071
Hypertension	318 (71.0)	363 (62.5)	0.004
Dyslipidemia	166 (37.1)	181 (31.2)	0.047
PVD	5 (1.1)	17 (2.9)	0.047
Prior MI	241 (53.8)	313 (53.9)	0.980
Prior PCI	80 (17.9)	114 (19.6)	0.473
Prior stroke	34 (7.6)	25 (4.3)	0.025
Heart failure	122 (27.2)	174 (29.9)	0.340
Systolic heart failure	54 (12.1)	81 (13.9)	0.374
Diastolic heart failure	70 (15.6)	94 (16.2)	0.810
CKD	8 (1.8)	7 (1.2)	0.441
COPD/asthma	1 (0.2)	6 (1.0)	0.146
Hyperuricemia	105 (23.4)	93 (16.0)	0.003
Smoking	228 (50.9)	272 (46.8)	0.195
Drinking	76 (17.0)	81 (13.9)	0.181
BMI (kg/m^2^)	26.53 (24.44–28.37)	26.42 (24.45–28.40)	0.397

Laboratory examination			
** **LVEF (%)	61.00 (57.00–67.00)	61.00 (56.00–66.00)	0.151
** **RWMA	131 (29.2)	180 (31.0)	0.547
** **Baseline fasting blood glucose (mmol/L)	6.90 (5.88–8.19)	8.11 (6.54–10.63)	≤0.001
** **Baseline HbA1c	7.13 (6.40–7.50)	7.80 (7.00–8.30)	≤0.001
** **Baseline HbA1c ≥ 7.0	222 (49.6)	437 (75.2)	≤0.001
** **Triglyceride (mg/dL)	1.61 (1.19–2.30)	1.61 (1.17–2.32)	0.965
** **HDL-C (mg/dL)	0.94 (0.82–1.07)	0.94 (0.82–1.10)	0.718
** **LDL-C (mg/dL)	2.31 (1.83–3.01)	2.34 (1.89–3.03)	0.421

Medical treatment			
** **Aspirin	443 (98.9)	569 (98.1)	0.316
** **P2Y_12_ inhibitor	399 (89.1)	521 (89.8)	0.692
** **Statin	415 (92.6)	553 (95.3)	0.066
** **Nitrites	174 (38.8)	253 (43.6)	0.123
** **Beta-blocker	350 (78.1)	447 (77.1)	0.688
** **CCB	121 (27.0)	152 (26.2)	0.773
** **ACEI/ARB	257 (57.4)	317 (54.7)	0.385
** **Insulin	120 (26.8)	253 (43.5)	≤0.001
** **Sulfonylureas	104 (23.2)	97 (16.7)	0.009
** **Nateglinide	31 (6.9)	28 (4.8)	0.151
** **Biguanides	181 (40.4)	238 (41.0)	0.856
** **Thiazolidinediones	89 (19.9)	87 (15.0)	0.039
** **Alpha-glucosidase inhibitor	179 (40.0)	226 (38.9)	0.731

Angiographic characteristics			
** **CTO location			
** **LAD	137 (30.6)	179 (30.8)	0.937
** **LCX	131 (29.2)	162 (27.9)	0.632
** **RCA	180 (40.2)	240 (41.3)	0.715

Number of diseased vessels			
** **1	124 (27.7)	129 (22.2)	0.043
** **2	180 (40.2)	218 (37.5)	0.386
** **3	144 (32.1)	234 (40.3)	0.007
Syntax score^#^	20.50 (17.00–25.50)	21.00 (18.25–26.50)	0.039
Rentrop grade ≥ 2^#^	322 (81.9)	422 (82.3)	0.899
Abrupt stump^#^	186 (47.3)	239 (46.6)	0.825
Calcification^#^	84 (21.4)	111 (21.6)	0.924
Bending ≥ 45°^#^	152 (38.7)	205 (40.0)	0.695
CTO length ≥ 20 mm^#^	190 (48.3)	277 (54.0)	0.092

Procedural characteristics			
** **Retrograde approach^*∗*^	32 (12.7)	32 (10.2)	0.349

Values are *n* (%), mean ± SD, or median with interquartile range. ACEI/ARB: angiotensin-converting enzyme inhibitor/angiotensin-receptor blocker; BMI: body mass index; CABG: coronary artery bypass grafting; CCB: calcium-channel blocker; CKD: chronic kidney disease; COPD: chronic obstructive pulmonary disease; CTO: chronic total occlusion; HCY: homocysteine; HDL-C: high-density lipoprotein cholesterol; HF: heart failure; LAD: left anterior descending coronary artery; LCX: left circumflex artery; LDL-C: low-density lipoprotein cholesterol; LVEF: left ventricular ejection fraction; MI: myocardial infarction; MT: medical therapy; PCI: percutaneous transluminal coronary intervention; PVD: peripheral vascular disease; RCA: right coronary artery; RWMA: regional wall motion abnormality; TC: total cholesterol. ^#^Cine angiogram records got from 906 (88.05%) individuals. ^*∗*^Only patients who were treated with PCI.

**Table 2:** Baseline characteristics in propensity score-matched population.

Clinical characteristics	HbA1c < 7.0 (*n* = 353)	HbA1c ≥ 7.0 (*n* = 353)	*P* value
Age (year)	59.28 ± 9.48	59.82 ± 9.67	0.458
Male	276 (78.2)	276 (78.2)	1.000
Hypertension	240 (68.0)	241 (68.3)	0.936
Dyslipidemia	116 (32.9)	130 (36.8)	0.269
PVD	5 (1.4)	10 (2.8)	0.192
Prior MI	184 (52.1)	184 (52.1)	1.000
Prior PCI	63 (17.8)	65 (18.4)	0.845
Prior stroke	15 (4.2)	19 (5.4)	0.482
Heart failure	93 (26.3)	106 (30.0)	0.277
Systolic heart failure	43 (12.2)	48 (13.6)	0.574
Diastolic heart failure	50 (14.2)	58 (16.4)	0.403
CKD	5 (1.4)	6 (1.7)	0.761
COPD/asthma	1 (0.3)	2 (0.6)	1.000
Hyperuricemia	70 (19.8)	71 (20.1)	0.925
Smoking	167 (47.3)	169 (47.9)	0.880
Drinking	51 (14.4)	46 (13.0)	0.585
BMI (kg/m^2^)	26.53 ± 3.14	26.49 ± 3.10	0.863

Laboratory examination			
LVEF (%)	61.00 (56.00–66.00)	61.00 (55.00–66.00)	0.692
RWMA	105 (29.7)	105 (29.7)	1.000
Baseline fasting blood glucose (mmol/L)	7.22 (6.09–8.50)	7.38 (6.15–9.15)	0.052
Baseline HbA1c	7.20 (6.70–7.60)	7.30 (6.80–7.90)	0.002
Baseline HbA1c ≥ 7.0	194 (54.96)	230 (65.16)	
Triglyceride (mg/dL)	1.63 (1.20–2.33)	1.63 (1.20–2.33)	0.865
HDL-C (mg/dL)	0.93 (0.81–1.05)	0.94 (0.82–1.10)	0.549
LDL-C (mg/dL)	2.31 (1.85–3.05)	2.34 (1.93–3.06)	0.270

Medical treatment			
Aspirin	348 (98.6)	344 (97.5)	0.280
P2Y_12_ inhibitor	317 (89.8)	316 (89.5)	0.902
Statin	333 (94.3)	334 (94.6)	0.869
Nitrites	140 (39.7)	141 (39.9)	0.939
Beta-blocker	270 (76.5)	271 (76.8)	0.929
CCB	92 (26.1)	85 (24.1)	0.543
ACEI/ARB	202 (57.2)	199 (56.4)	0.820
Insulin	110 (31.2)	130 (36.8)	0.112
Sulfonylureas	78 (22.1)	64 (18.1)	0.189
Nateglinide	22 (6.2)	21 (5.9)	0.875
Biguanides	140 (39.7)	145 (41.1)	0.701
Thiazolidinediones	62 (17.6)	58 (16.4)	0.689
Alpha-glucosidase inhibitor	146 (41.4)	134 (38.0)	0.356

Angiographic characteristics			
CTO location			
LAD	112 (31.7)	110 (31.2)	0.871
LCX	99 (28.0)	88 (24.9)	0.348
RCA	142 (40.2)	155 (43.9)	0.322

Number of diseased vessels			
1	93 (26.3)	90 (25.5)	0.797
2	138 (39.1)	136 (38.5)	0.877
3	122 (34.6)	127 (36.0)	0.694
Syntax score^#^	21.00 (17.50–26.50)	21.00 (17.50–24.50)	0.766
Rentrop grade ≥ 2^#^	257 (83.2)	256 (82.6)	0.845
Abrupt stump^#^	152 (49.2)	155 (50.0)	0.840
Calcification^#^	66 (21.4)	73 (23.5)	0.514
Bending ≥ 45°^#^	119 (38.5)	130 (41.9)	0.385
CTO length ≥ 20 mm^#^	158 (51.1)	171 (55.2)	0.315

Procedural characteristics			
Retrograde approach^*∗*^	29 (15.0)	22 (11.5)	0.302

Values are *n* (%), mean ± SD, or median with interquartile range. ACEI/ARB: angiotensin-converting enzyme inhibitor/angiotensin-receptor blocker; BMI: body mass index; CABG: coronary artery bypass grafting; CCB: calcium-channel blocker; CKD: chronic kidney disease; COPD: chronic obstructive pulmonary disease; CTO: chronic total occlusion; HCY: homocysteine; HDL-C: high-density lipoprotein cholesterol; HF: heart failure; LAD: left anterior descending coronary artery; LCX: left circumflex artery; LDL-C: low-density lipoprotein cholesterol; LVEF: left ventricular ejection fraction; MI: myocardial infarction; MT: medical therapy; PCI: percutaneous transluminal coronary intervention; PVD: peripheral vascular disease; RCA: right coronary artery; RWMA: regional wall motion abnormality; TC: total cholesterol. ^#^Cine angiogram records got from 619 (87.68%) individuals. ^*∗*^Only patients who were treated with PCI.

**Table 3:** Estimated Kaplan–Meier events rates in propensity score-matched population.

	HbA1c < 7.0	HbA1c ≥ 7.0	Unadjusted HR (95% CI)	Adjusted HR (95% CI)
MACE	119 (33.7)	140 (39.7)	1.422 (1.027–1.970)	1.531 (1.009–2.149)
Cardiac death	17 (4.8)	15 (4.2)	0.913 (0.434–1.921)	0.717 (0.324–1.584)
Repeat revascularization	99 (28.0)	123 (34.8)	1.618 (1.111–2.356)	1.828 (1.238–2.698)
Repeat nonfatal MI	21 (5.9)	16 (4.5)	0.582 (0.268–1.261)	0.513 (0.235–1.119)
All-cause death	24 (6.8)	23 (6.5)	1.044 (0.561–1.943)	0.878 (0.457–1.687)
TVR	57 (16.1)	76 (21.5)	1.595 (0.957–2.657)	1.668 (0.994–2.796)

Adjusted covariates: age, CKD, LAD-CTO, LCX-CTO, LVEF, PVD, Rentrop grade ≥ 2, RWMA, sex, single-vessel disease, systolic heart failure, SYNTAX score, and triple-vessel disease. CKD: chronic kidney disease; CI: conference interval; HR: hazard ratio; LAD-CTO: left anterior descending artery-chronic total occlusion; LCX-CTO: left circumflex chronic total occlusion; LVEF: left ventricular ejection fraction; MACE: major adverse cardiac events, a composite of cardiac death, repeat revascularization, and repeat nonfatal MI; MI: myocardial infarction; PVD: peripheral vascular disease; RWMA: regional wall motion abnormalities; SYNTAX: percutaneous coronary intervention with taxus and cardiac surgery; TVR: target vessel revascularization.

**Table 4:** Estimated Kaplan–Meier event rates in subgroups of propensity score-matched population.

	CTO-SR				CTO-NSR			
	HbA1c < 7.0	HbA1c ≥ 7.0	Unadjusted HR (95% CI)	Adjusted HR (95% CI)	HbA1c < 7.0	HbA1c ≥ 7.0	Unadjusted HR (95% CI)	Adjusted HR (95% CI)
MACE	53 (27.7)	63 (32.3)	1.254 (0.781–2.015)	1.284 (0.784–2.102)	66 (40.7)	77 (48.7)	1.566 (0.996–2.462)	1.826 (1.112–2.999)
Cardiac death	9 (4.7)	7 (3.6)	0.650 (0.210–2.011)	0.581 (0.172–1.959)	8 (4.9)	8 (5.1)	1.165 (0.422–3.216)	1.514 (0.422–5.433)
Repeat revascularization	43 (22.5)	56 (28.7)	1.601 (0.926–2.766)	1.730 (0.980–3.055)	56 (34.6)	67 (42.4)	1.627 (0.969–2.733)	1.906 (1.091–3.330)
Repeat nonfatal MI	8 (4.2)	8 (4.1)	0.935 (0.314–2.785)	0.992 (0.323–3.043)	13 (8.0)	8 (5.1)	0.351 (0.112–1.103)	0.242 (0.072–0.817)
All-cause death	12 (6.3)	10 (5.1)	0.738 (0.279–1.952)	0.804 (0.288–2.248)	12 (7.4)	13 (8.2)	1.321 (0.579–3.015)	1.133 (0.425–3.022)
TVR	22 (11.5)	31 (15.9)	1.207 (0.567–2.570)	1.342 (0.590–3.052)	35 (21.6)	45 (28.5)	1.995 (0.987–4.034)	2.194 (1.059–4.548)

Adjusted covariates: age, CKD, LAD-CTO, LCX-CTO, LVEF, PVD, Rentrop grade ≥ 2, RWMA, sex, single-vessel disease, systolic heart failure, SYNTAX score, and triple-vessel disease. CKD: chronic kidney disease; CI: conference interval; HR: hazard ratio; LAD-CTO: left anterior descending artery-chronic total occlusion; LCX-CTO: left circumflex chronic total occlusion; LVEF: left ventricular ejection fraction; MACE: major adverse cardiac events, a composite of cardiac death, repeat revascularization, and repeat nonfatal MI; PVD: peripheral vascular disease; RWMA: regional wall motion abnormalities; SYNTAX: percutaneous coronary intervention with taxus and cardiac surgery; TVR: target vessel revascularization.

## Data Availability

The data sets generated and analyzed for this current study are available from the corresponding author upon reasonable request.

## Abbreviations

CTO: Chronic total occlusion
HbA1c: Glycosylated hemoglobin A
CTO-SR: Successful CTO revascularization
CTO-NSR: Nonsuccessful CTO revascularization
MACE: Major adverse cardiovascular events
MI: Myocardial infarction
TVR: Target vessel revascularization
PCI: Percutaneous coronary intervention
CABG: Coronary artery bypass grafting
HIS: Hospital information system
TIMI: Thrombolysis in myocardial infarction
ARC: Academic Research Consortium
HRs: Hazard ratios
CKD: Chronic kidney disease
PVD: Peripheral vascular disease
LVEF: Left ventricular ejection fraction
RWMA: Regional wall motion abnormalities
LAD-CTO: Left anterior descending artery-CTO
LCX-CTO: Left circumflex-CTO
SYNTAX: Percutaneous coronary intervention with taxus and cardiac surgery
ASDs: Absolute standardized differences
VADT: Veterans affairs diabetes trial
DM: Diabetes mellitus
EES: Everolimus-eluting stents.
